# ^99m^Tc-DMSA (V) in Evaluation of Osteosarcoma: Comparative Studies with ^18^F-FDG PET/CT in Detection of Primary and Malignant Lesions

**DOI:** 10.5402/2012/371830

**Published:** 2012-05-07

**Authors:** G. P. Bandopadhyaya, Priyanka Gupta, Archana Singh, Jaya Shukla, S. Rastogi, Rakesh Kumar, Arun Malhotra

**Affiliations:** ^1^Department of Nuclear Medicine, All India Institute of Medical Sciences, New Delhi 110029, India; ^2^Department of Nuclear Medicine, Post Graduate Institute of Medical Education and Research, Chandigarh 160012, India; ^3^Department of Orthopaedics, All India Institute of Medical Sciences, New Delhi 110029, India

## Abstract

To evaluate the role of ^99m^Tc-DMSA (V) and [^18^F]FDG PET-CT in management of patients with osteosarcoma, 22 patients were included in our study. All patients underwent both ^99m^Tc-DMSA (V) and whole-body [^18^F]FDG PET-CT scans within an interval of 1 week. 555–740 MBq of ^99m^Tc-DMSA (V) was injected i.v. the whole-body planar, SPECT images of primary site and chest were performed after 3-4 hours. [^18^F]FDG PET-CT images were obtained 60 minutes after i.v. injection of 370 MBq of F-18 FDG. Both FDG PET-CT (mean SUV_max_ = 7.1) and DMSA (V) scans showed abnormal uptake at primary site in all the 22 patients (100% sensitivity for both). Whole-body PET-CT detected metastasis in 11 pts (lung mets in 10 and lung + bone mets in 1 patient). Whole-body planar DMSA (V) and SPECT detected bone metastasis in one patient, lung mets in 7 patients and LN in 1 patient. HRCT of chest confirmed lung mets in 10 patients and inflammatory lesion in one patient. 7 patients positive for mets on DMSA (V) scan had higher uptake in lung lesions as compared to FDG uptake on PET-CT. Three patients who did not show any DMSA uptake had subcentimeter lung nodule. Resuts of both ^99m^Tc-DMSA (V) (whole-body planar and SPECT imaging) and [^18^F]FDG PET-CT were comparable in evaluation of primary site lesions and metastatic lesions greater than 1 cm. Though ^99m^Tc-DMSA (V) had higher uptake in the lesions as compared to [^18^F]FDG PET-CT, the only advantage [^18^F]FDG PET-CT had was that it could also detect subcentimeter lesions.

## 1. Introduction

OS is a primary malignant bone tumor characterized by the direct formation of immature bone or osteoid tissue by the tumor cells. It is thought to arise from primitive mesenchymal bone-forming cells. The classic OS is a rare (45% of all malignant bone tumors) highly malignant tumor [1, 2], with an estimated incidence of 3 cases/million population/year. OS arises predominantly in the metaphysis of long bones, the most common sites of involvement are femur (42%, 75% of which are distal femur), tibia (19%, 80% of which are proximal tibia), and humerus (10%, 90% of which are proximal humerus). Other significant locations are the skull and jaw (8%) and pelvis (8%). The age at presentation ranges from 10 to 25 years of age.

The pretherapeutic diagnostic workup usually involves physical examination, Radiological examination (plain radiograph, computed tomography, magnetic resonance imaging, angiography), radionuclide imaging (bone scintigraphy, ^99m^Tc-DMSA (V), ^18^F-FDG-PET imaging), biopsy and lab tests.

The aim of our study was to evaluate the role of Pentavalent ^99m^Tc-Dimercaptosuccinic acid [^99m^Tc-DMSA (V)] and ^18^F-Fluoro-2-Deoxy-Glucose ^18^F-FDG PET-CT in the management of patients with osteosarcoma and to compare results of ^99m^Tc-DMSA (V) with ^18^F-FDG PET-CT scan in these patients.

## 2. Materials and Methods

A total of 22 patients (14 males and 8 females) with biopsy-proven OS were included in this study. Age of the patients ranged from 8–66 yrs (mean age = 22.2 yrs). Pre- or postchemotherapy patients (both the types of patients) before surgery were included. Patients excluded were those who had undergone surgery before the nuclear medicine examinations and patients with diabetes mellitus or pathologic glucose tolerance. The patients with primary metastasis disease were not excluded.

### 2.1. Radiopharmaceuticals

We used two types of imaging modalities with two different radiopharmaceuticals, ^99m^Tc-DMSA (V) for whole-body scintigraphy using gamma camera and ^18^F-FDG for PET-CT imaging. ^99m^Tc is a gamma emitter and generator produced while ^18^F is a positron emitter and cyclotron produced.

### 2.2. PET/CT Data Acquisition Protocol

After fasting for at least 6 hrs, verifying the serum glucose level (that should be below 140 mg/dL) and with patients in a resting state, in a quiet room, a dose of 5–10 mCi of ^18^F-FDG was injected intravenously depending on the age and weight of the patient. After a 45–60 minutes uptake period, patient was placed into the scanner. Initial CT acquisition was done without oral or intravenous contrast injection; followed by PET scan. On the PET/CT scanner, CT scan acquisition was performed on spiral dual slice CT with a slice thickness of 4 mm and a pitch of 1. Images were acquired using a matrix of 512 × 512 pixels and pixel size of about 1 mm. After completing the CT, the table is moved towards the field of view of PET. After transmissions scan, 3D PET acquisition was taken for 3–5 minutes per bed position for 5–7 bed positions depending on the patient length. PET data was acquired using a matrix of 128 × 128 pixels, and PET acquisition of the same axial range, as was with CT, was taken with the patient in the same position on the table. Separate scans of the primary site were taken if it was not coming in the FOV of the whole-body PET-CT acquisition. CT-based attenuation correction of the emission images was done.

## 3. Processing Protocol

The CT images were acquired and reconstructed using optimized parameters for attenuation correction. Data obtained from the CT acquisition was used for low noise attenuation correction of PET emission data and for fusion of attenuation corrected PET images with the corresponding CT images. After completion of PET acquisition, images were reconstructed by iterative method ordered subset expectation maximization (2 iterations and 8 subsets) with a filter of 5 mm. The reconstructed attenuation—corrected PET images, CT images and fused images of matching pairs of PET and CT images—was available for review in axial, coronal, and sagittal planes and in maximum intensity projections, three dimensional cine mode.

After image reconstruction, a region of interest (ROI) was carefully drawn around the site of the abnormal FDG uptake on lesion/s in the consequent 4–6 PET-CT scan slices. The slice with a maximal FDG uptake in the ROI was chosen for quantitative measurement of metabolic activity of the tracer (SUV). From these ROIs, the SUV was calculated according to the formula described below:

(1) $$\text{SUV}=\frac{\text{Mean}\;\;\text{ROI}\;\;\text{activity}\;\;\left(\text{MBq}/\text{g}\right)}{\text{Injected}\;\;\text{dose}\;\;\left(\text{MBq}\right)/\text{Body}\;\;\text{weight}\;\;\left(\text{g}\right)},$$

whereas “MBq” is the mega-Becquerel and “g” is the grams.

### 3.1. ^99m^Tc-DMSA (V) Scanning

Patients were injected with 15–20 mCi of ^99m^Tc-DMSA (V), depending upon the age and weight of the patients. Dual head gamma camera was used and whole-body scans were taken 3-4 hours after the injection. SPECT images of the primary and the chest were also taken. Spot views were also taken if needed.

### 3.2. Acquisition Protocol

Whole-body planar images were acquired using the continuous mode with a table speed of 20 cms/sec from head to toe. A matrix size of 256 × 1024 was used. The photopeak was kept at 140 keV with a 20% energy window. Scan lengths were taken according to the length of the patients.

**SPECT Images of the Primary Site and Chest Region were also Taken**

SPECT images were taken with the following protocol.

matrix size: 64 × 64,
frame time: 15–18 sec/frame,
angular rotation: 3°/frame,
circular orbit: 360°.

### 3.3. Data Interpretation

Two experienced nuclear medicine physicians evaluated the scan findings independently and they were blinded to the structural imaging findings and clinical findings. Images were looked for area of increased radiotracer uptake and corresponding areas in the CT images, and fused PET-CT images were corroborated.

### 3.4. Statistical Analysis

Multiple regression analysis was performed to find the significance of correlation (*r* > 0.5 and *P* < 0.05 were considered significant). The intensity and the extent of uptake in ^18^F-FDG-PET-CT versus ^99m^Tc-DMSA (V) at the primary site were analysed quantitatively and qualitatively. Accompanying lung lesions absent versus present and their uptake imperceptible versus perceptible were compared visually in both the modalities.

## 4. Results

**Figure 1:**
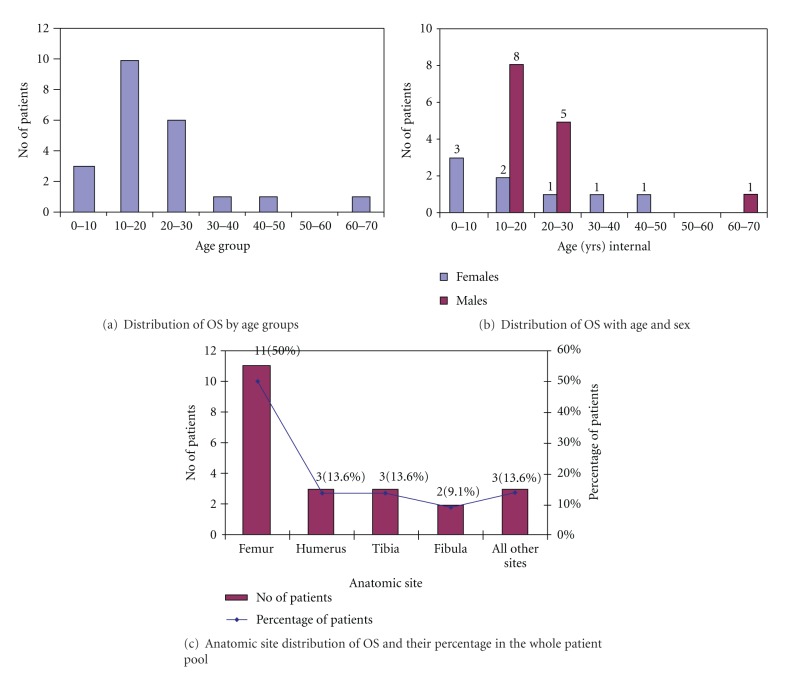
Graphs showing distribution of OS by age, sex, and anatomic site of distribution.

In this prospective study a total of 22 patients were included. All the patients were already biopsy-proven cases of OS. All of them underwent thoracic CT within the two weeks before or after ^18^F-FDG-PET/CT and ^99m^Tc-DMSA (V) scan. Those patients who were taken after chemotherapy had undergone therapy at least two weeks before the ^18^F-FDG-PET/CT and ^99m^Tc-DMSA (V) scan. Characteristics of all patients are given in Table 1 with graphical representation in Figure 1.

**Age**

In 22 patients considered for this study, range of age was 8–66 years, with a mean age of 22.2 years. Maximum number of patients falls within the range of 10–20 years, with 10 patients (45.5%) falling in this interval. Out of these patients maximum number of patients as males 8/10 (80%) and only 2/10 (20%) were females. Next higher number of patients, as in the range of 20–30 years with 6/22 patients (27.3%), including 5/6 (83%) males and 1/6 females (16.7%). Interval of 1–10 years also included 3 patients (13.6%); all were >5 years in age with 100% females. Interval 30–40 years and 40–50 years included 1 patient (4.5%) each and both were females. 50–60 years of interval included no patient. Another interval of 60–70 years included 1 patient (4.5%), who was male.

**Sex**

Out of 22 patients, 14 patients (63.6%) were males, 8 were females (36.4%). Male- to- female ratio was 1.74. Out of 14 males, maximum number of males was in the age group of 10–20 years. In higher age groups of 30–50, 2 females (9%) out of 22 patients were diagnosed with OS.

### 4.1. Anatomic Site Distribution of OS

Of 22 patients, 11 patients had femur as primary site (10 cases (90%)  of distal femur and 1/11 patient (10%) of proximal femur), 3 patients had humerus as primary site (with 100% proximal humerus involvement), and 3 patient had tibial involvement of OS (all the cases of proximal Tibia). 2 patients (9.1%) were having fibula inclusion (100% proximal fibula). Other anatomic locations were also diagnosed. Three patients were having primary site of OS other than extremities. Out of these 3 (13.6%), 1 had chest involvement (patient 6), another patient had OS in clavicle (patient 11) and the last one was with jaw involvement (patient 22) with each having a percentage contribution of 4.5% to all the patients (Table 2).

### 4.2. ^18^F-FDG-PET-CT Findings

#### 4.2.1. Primary Site

**Figure 2:**
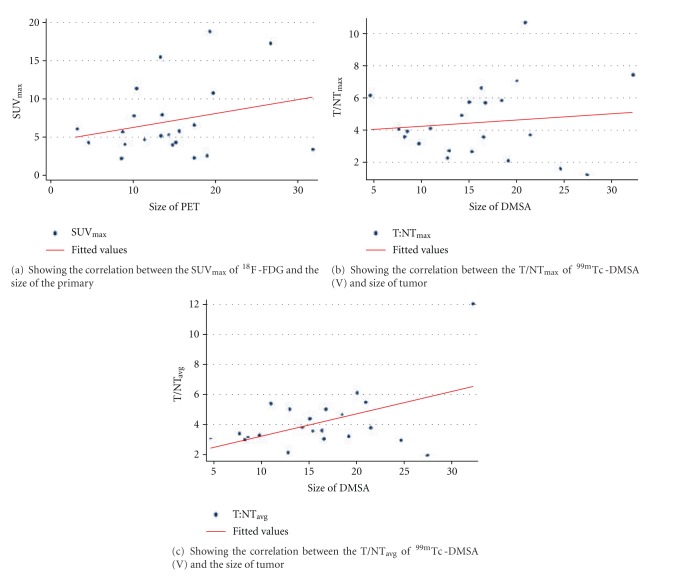
Graphs showing correlation between SUV_max_ of ^18^F-FDG with size of tumour and between T/NT_max_ and T/NT_avg_ with size of tumour.

In all 22 patients, a baseline ^18^F-FDG-PET-CT examination was performed without any chemotherapy or after a minimum of two weeks of chemotherapy; the primary or residual tumor site demonstrated a clear or moderate increased glucose uptake. Abnormal FDG uptake was seen at the primary site in all the 22 patients showing the involvement of the primary site. ^18^F-FDG-PET-CT showed a mean of SUV_max⁡_ = 7.104 ± 4.7 (range of SUV_max⁡_ = 2.2–18.9) of the primary site. The details of individual ^18^F-FDG uptake are given in all patients in Table 3. Since they were already histopathologically proven, so the sensitivity of ^18^F-FDG-PET-CT was 100% in detecting the primary site. No correlation was found between the size of the tumor and the SUV_max_ (*r* = 0.1209) (Figure 2(a)).

#### 4.2.2. Metastases

Analyzing the results of whole-body ^18^F-FDG-PET-CT scans each patient underwent, foci with increased pulmonary/pleural tracer uptake was found in 11/22 patients. By correlating clinical and other imaging findings, 10/11 (90%) of the examinations were classified as true positive, one as false positive. Out of 11 patients, 8 had bilateral lung nodule with variable degree of FDG uptake (mean SUV_max_ = 1.1). One patient (patient 9) had bilateral lung nodule with no FDG uptake in one nodule. The other remaining two patients had solitary lung nodules (patients 1, 21). Out of these 2, “1” had mild [^18^F]FDG uptake while the other found to be inflammatory lesion on HRCT (patient 21). So ^18^F-FDG-PET-CT was found to have a sensitivity of 100% in detecting the lung mets and specificity of 91% in comparison to the other clinical and imaging modalities findings. Although PET alone was not able to detect many lesions but with the incorporation of CT, sensitivity of PET increased tremendously.

With regard to other metastatic locations, abnormal uptake was also detected in one patient (patient 10) at the site of left scapula (coracoid process). No other abnormal uptake was detected at any other site.

### 4.3. ^99m^Tc-DMSA (V) Scan Findings

All the 22 patients underwent ^99m^Tc-DMSA (V) scanning within one week, before or after, ^18^F-FDG-PET-CT scans.

#### 4.3.1. Primary Site

Whole-Body planar imaging and SPECT images of the primary site of OS in all the patients revealed variable degree of uptake of ^99m^Tc-DMSA (V) at the primary tumor site. Large variation was seen in the tumor-to-nontumor ratios (T/NTs). The mean of maximum T/NT or T/NT_max_ Was Found to be 4.446 ± 2.27 (Ranging from 1.148–10.689). The mean value of T/NT_avg_ came out to be 4.125 (ranging from 1.881–12.01). Individual patient values of T/NT_max_ and T/NT_avg_ are given in Table 3. So ^99m^Tc-DMSA (V) showed 100% sensitivity in detecting the primary site like ^18^F-FDG-PET-CT findings. t/nts of ^99m^Tc-DMSA (V) showed no statistically significant correlation with spect to the size of the primary tumor. T/NT_max_ (**r** = 0.0311) (Figure 2(b)) and T/NT_avg_ (**r** = 0.2490) (Figure 2(c)) were nowhere correlated to the size of the tumor. increased blood pool activity was evident in all the scans, which decreased with the delayed imaging.

#### 4.3.2. Metastases

Spect of Chest in all the patients were taken along with the whole body scans to detect the deep seated metastasis. of all the 10/22 patients already proved to have lung metastasis 7 patients (70%) showed abnormal uptake in the lungs. increased uptake of ^99m^Tc-DMSA (V) could be easily identified as focal, sharply defined regions with high lesion-to-background ratio (L/N). the mean of maximum lesion-to-background ratio (L/N_max_) was 1.8154 (1.5–20.23) with patient 4 showing the highest value of 20.23. the mean of average lesion-to-background ratio (L/N_avg_) was found to be 1.1987 (1.298–12.166) in 6 out of 7 patients. Intensity of ^99m^Tc-DMSA (V) uptake was either more (Figure 6) or comparable to the fdg uptake in ^18^F-FDG-PET-CT, while one patient (patient 16) showed less uptake in comparison to ^18^F-FDG-PET-CT. patient 16 had large number of nodules with intense fdg uptake on ^18^F-FDG-PET scan while ^99m^Tc-DMSA (V) showed only one lesion with less intensity of uptake. Lesions in rest of the 3 patients were not detected on the ^99m^Tc-DMSA (V) scan. 3/10 patients (33%) (patient 1, 9, 10) who did not show any ^99m^Tc-DMSA (V) uptake either had sub centimeter lung nodule (patient 1 and 9) which because of the limited resolution of gamma camera (0.9–1.3 cm) and the increased blood pool activity of ^99m^Tc-DMSA (V) in the major cardiovascular structures, were not visible in spect or was post chemotherapy (patient 10) who showed significant response to the chemotherapy. also the overall number of lung lesions detected with ^99m^Tc-DMSA (V) was less in all the 7 patients.

#### 4.3.3. Other Sites

Abnormal uptake was also detected in patient 10 in the left scapula region, as was seen in the ^18^F-FDG-PET-CT images ^99m^Tc-DMSA (V) could also show the amount of necrosis and soft tissue involvement at primary site. Patient 9 also showed uptake in the right axillary lymph node. In case of children, there was an increased uptake at the growth plates because of increased growth at these points (patients 3, 5, 7, 11, 14, 16, 17, and 20).

#### 4.3.4. SUV_max_ of ^18^F-FDG-PET-CT and T/NTs of   ^99m^Tc-DMSA (V)

**Figure 3:**
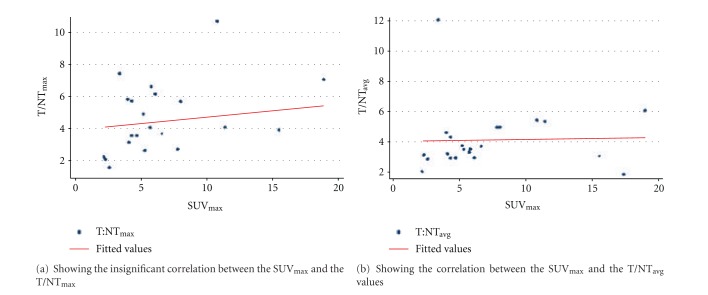
Graphs showing correlation between SUV_max_ and T/NT_max_ and between SUV_max_ and T/NT_avg_.

No statistically significant correlation was found between SUV_max_ of ^18^F-FDG-PET-CT and the T/NTs ratios of ^99m^Tc-DMSA (V) of primary site with SUV_max_ and T/NT_max_ (*r* = 0.2711, *P* = 0.2223), and SUV_max_ and T/NT_avg_ (*r* = 0.30, *P* = 0.18) (Figures 3(a) and 3(b)).

## 5. Discussion

OS is a rare form of bone cancers, which mainly affects the people in the age group of 10–25 years [3]. The 5-year relative survival for children with bone cancer has improved from 49% to 63% in the recent time with the development of improved diagnostic modalities, which helps in the early detection of disease. Early detection of the disease has good prognosis. It usually metastasizes to the lungs and bones which is associated with poor prognosis. So it is essential to know about the metastatic involvement of the disease. In the present study we tried to assess the role of ^99m^Tc-DMSA (V) in detecting the primary as well as the metastatic involvement of the OS by comparing it with the ^18^F-FDG-PET-CT.

Role of ^18^F-FDG-PET in characterizing the tumor as malignant and benign has already been studied extensively and it was found to be a promising tool to study the nature of tumor noninvasively [4–7]. ^99m^Tc-DMSA (V), which is a tumor targeting radiopharmaceutical, has also found application in detection of medullary thyroid carcinoma, amyloidosis, head and neck cancers, and soft tissue tumors. It was found to have higher sensitivity in detecting the bone metastasis and soft tissue tumors than ^99m^Tc-MDP [8, 9]. No study has been done till now to compare the role of ^99m^Tc-DMSA (V) and ^18^F-FDG-PET-CT in imaging the primary tumor of OS. However, one study which compared the ^99m^Tc-DMSA (V), ^99m^Tc-MDP, and CT found the scans of ^99m^Tc-DMSA (V) superior to the ^99m^Tc-MDP scans and the CT scans in identifying the metastases of OS [10].

**Figure 4:**
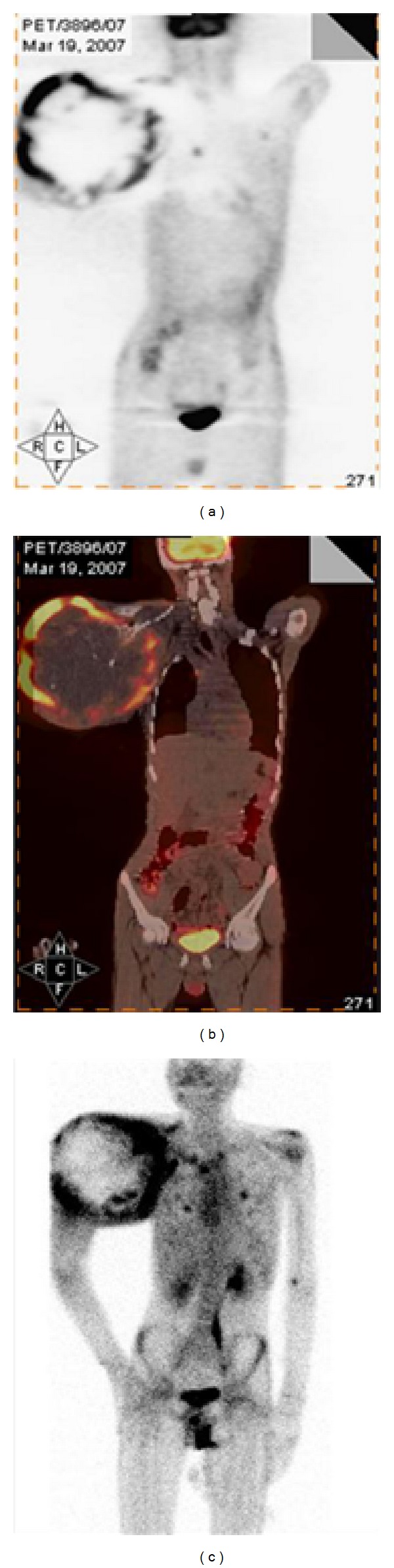
Showing intense [^18^F]FDG uptake (a) and the ^99m^Tc-DMSA (V) uptake (c) at the primary site of Rt humerus.

**Figure 5:**
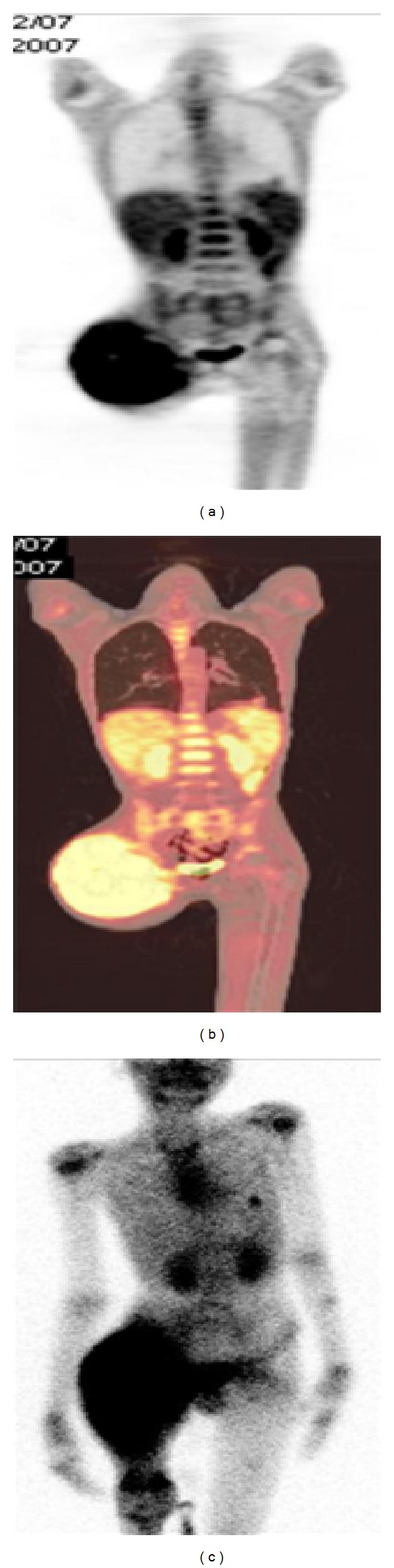
Intense uptake of [^18^F]FDG (a) and ^99m^Tc-DMSA (V) (c) is seen at the primary site in the Rt femur along with the lung nodule in the left lung lower lobe.

**Figure 6:**
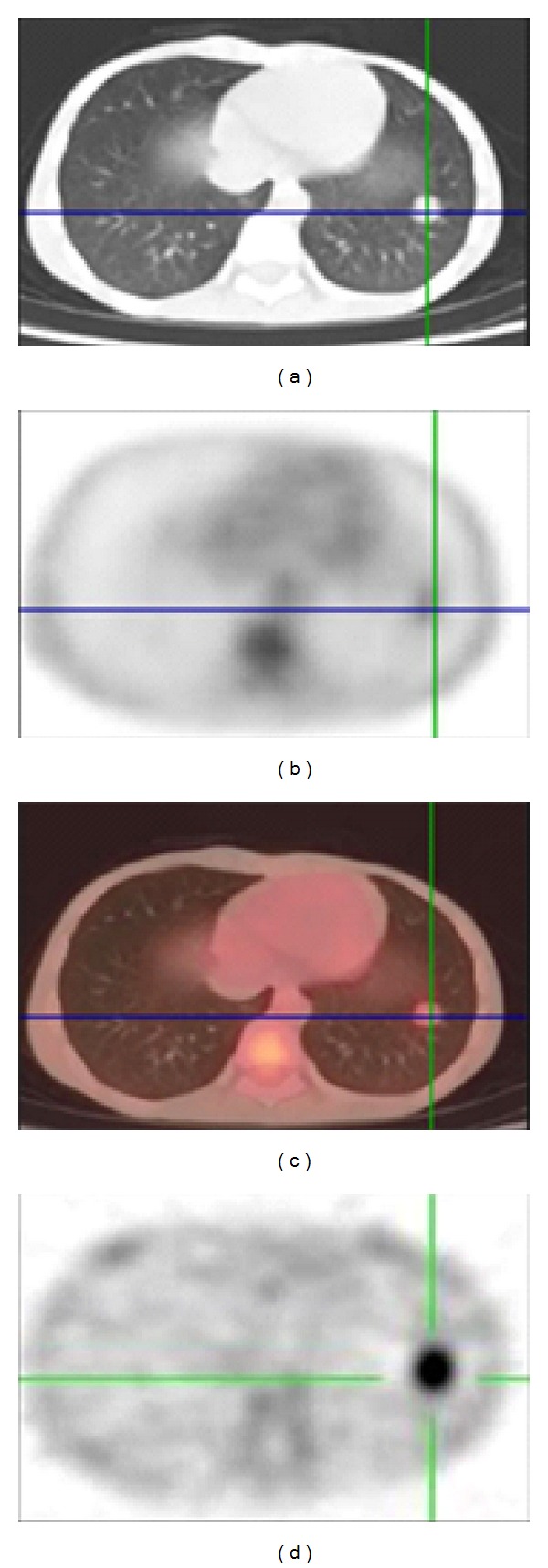
Showing the intense uptake in the left lung lower lobe nodule with higher intensity of uptake on ^99m^Tc-DMSA (V) (d) than [^18^F]FDG.

**Figure 7:**
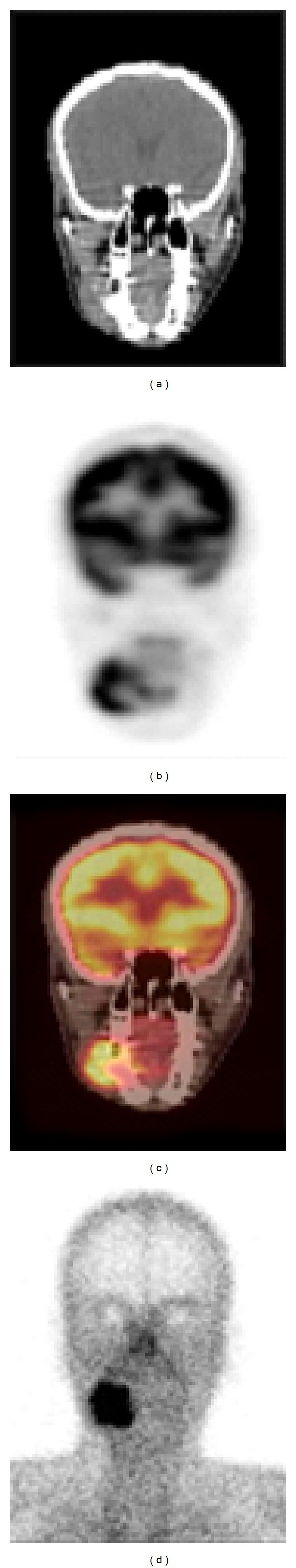
Showing the uptake of both the [^18^F]FDG (b) and the ^99m^Tc-DMSA (V) (d) in the primary at Rt jaw.

In our study we found that both the ^18^F-FDG-PET-CT and ^99m^Tc-DMSA (V) showed intense uptake at the primary site in all the patients (Figures 4, 5, and 7). ^99m^Tc-DMSA (V) showed equal sensitivity to ^18^F-FDG-PET in detecting primary tumor sites. The extent of localization and the intensity of uptake were comparable in both the cases. Although no significant statistical correlation was found between the SUV_max_ and T/NT ratios of ^18^F-FDG and ^99m^Tc-DMSA (V), respectively. But the detection efficiency of the two modalities was same. We found in our study that there is no significant statistical correlation between the SUV_max_ or the T/NT ratios and the size of the primary tumors. This could be due to the association of primary tumors of OS with extensive tumor necrosis and calcification which causes the nonuniform uptake of radiopharmaceutical.

^18^F-FDG-PET has also shown a higher sensitivity (98%) in detecting lung mets, equivalent to CT. A positive ^18^F-FDG-PET result can be used to confirm any abnormalities seen on thoracic CT scans as metastatic [11]. In our study ^18^F-FDG-PET-CT showed 100% sensitivity in detecting the lung mets. In case of ^99m^Tc-DMSA (V) only 7 patients were found to have lung mets. The decreased sensitivity of ^99m^Tc-DMSA (V) could be attributed to the limited resolution of gamma camera (0.9–1.3 cm) as most of the nodules, which were not seen, were subcentimetre in size.

However, PET alone showed abnormal lung uptake in 8 patients only out of 10 proved patients with lung mets. This could be due to the partial-volume averaging effect (which is a reduction in detected activity that occurs when the object size is smaller than the axial or transaxial spacing) [12]. The signal of small lesions could be “diluted” during reconstruction and malignant lesions can result in false negative findings on PET images. Effect of respiratory motion is also well known to cause artifacts on PET images. Although with the introduction of PET-CT, its sensitivity has improved tremendously with the anatomic localization of various structures especially in case of evaluation of childhood sarcomas [13]. Similarly detection efficiency of ^99m^Tc-DMSA (V) can also be enhanced with the incorporation of SPECT-CT.

In most of the cases, uptake in the normal bone was not detectably greater than the surrounding soft tissue. In the entire patients, both the excretion and retention in kidneys were apparent, with excreted radioactivity exclusively in the form of ^99m^Tc-DMSA (V) [9], but the degree of retention was variable. No area of increased uptake was noted in the thyroid gland. Few females showed increased breast uptake. Increased uptake in the liver was also seen in few patients. Although no correlation was found between the extent of disease and the renal and hepatic uptake, no correlation could also be established between the extent of metastasis and renal and liver uptake.

^18^F-FDG-PET has a disadvantage of showing a high number of false positive results because of the increased uptake in many inflammatory and benign nodules (as we had one patient which had false positive uptake in the inflammatory fibronodular lesion on ^18^F-FDG scan). Tumor metabolism and, consequently tumor detection with ^18^F-FDG-PET, may be highly susceptible to chemotherapy. While ^99m^Tc-DMSA (V) is a tumor seeking agent and is not metabolism dependent the extent of tumor uptake can differentiate malignant and chondrogenic tumors. ^99m^Tc-DMSA (V) has an advantage of detecting bone as well as soft tissue tumor [9, 14]. Although several instances of false-positive uptake have occurred in both the soft-tissue as well as skeletal disease but the lesions latter showed relatively low uptake on bone scans. However, no false positive result was obtained in our study with ^99m^Tc-DMSA (V). We could also detect abnormal uptake in bone mets at the scapula by both the imaging modalities.

Apart from application in imaging tumors, interest in the biodistribution of ^99m^Tc-DMSA (V) is increasing these days. Since the **β**-emitting analogues 186/188ReDMSA (V) offer the potential for targeted radiotherapy, the avidity with which the tracer is taken up in most bone metastasis suggests that this could be applied to palliative treatment [15].

Being a technetium labeled compound, ^99m^Tc-DMSA (V) is more economical and more accessible in comparison to the ^18^F-FDG-PET-CT. There is no report of any false positive uptake anywhere else in our study, which further increases its reliability. So the use of ^99m^Tc-DMSA (V) can be thought over for scanning the patients with OS in detecting the primary as well as the metastatic involvement at least in those areas where ^18^F-FDG-PET-CT is not available.

## 6. Conclusion

Pentavalent ^99m^TcDimercaptosuccinic acid appears to be a promising radiopharmaceutical for detecting primary and metastases in patients with osteosarcoma. ^18^F-FDG PET-CT also plays an important role in detecting primary and metastases in these patients. When ^99m^Tc-DMSA (V) is compared with 18F-FDG-PET-CT, it has lower sensitivity in detecting smaller metastatic lesions due to lower spatial resolution of gamma camera as compared to PET-CT. However, it requires costly setup including cyclotron, 18F-FDG synthesizing cell and a PET/CT camera, thereby the overall cost of an ^18^F-FDG-PET/CT scan is also more, which may be as much as 15 to 20 times the cost of a ^99m^Tc- DMSA (V) scan. Although ^99m^Tc-DMSA (V) was not as sensitive as ^18^F-FDG-PET-CT was, in detection of subcentimetre nodules, but it seems to be an good alternative to ^18^F-FDG-PET/CT in those areas where PET/CT is not available. Also, the sensitivity of ^99m^Tc-DMSA (V) scan may be improved for detection of sub centimeter nodules by using the SPECT-CT, which further improves the anatomical location, keeping the cost of scan still significantly less compared to an ^18^F-FDG-PET/CT scan.

^99m^Tc-DMSA (V) also has another advantage over ^18^F-FDG-PET/CT. The average whole-body dose delivered to the patient in a ^99m^Tc-DMSA (V) is found to be approximately 3mSv [16], while in a ^18^F-FDG-PET/CT scan, the average dose delivered has been found to be 11-12 mSv. Thus a ^99m^Tc-DMSA (V) scan delivers 4 times less radiation dose to the patient as compared to a ^18^F-FDG-PET/CT scan.

## Figures and Tables

**Table 1:** Patient's characteristics.

Characteristics	No. of patients (%)
Total no. of patients	22

Sex	
(i) Male	14 (63.6%)
(ii) Female	8 (36.4%)

Age	
(i) Age range	8–66 years
(ii) Mean age	21.55 years

Prechemotherapy	19
Postchemotherapy	3

Primary site of osteosarcoma	
(i) Femur	11 (50%)
(ii) Humerus	3 (13.6%)
(iii) Tibia	3 (13.6%)
(iv) Fibula	2 (9.1%)

Other sites	
(i) Chest	1 (4.5%)
(ii) Clavicle	1 (4.5%)
(iii) Jaw	1 (4.5%)

No. of patients with lung metastasis on CT	10

No. of patients having distant metastases	1

**Table 2:** Individual patient's characteristics describing the age, sex, primary site involved, histopathological subtype, and metastatic involvement of lung or any other site.

Patient no.	Age (y)	Sex	Primary site	Subtype	Lung mets	Other sites
1	48	F	Rt Humerus	N/A	P	N/I
2	16	M	Rt Tibia	MMT	P	N/I
3	13	F	Rt Femur	N/A	N	N/I
4	17	M	Rt Humerus	N/A	P	N/I
5	13	M	Rt Femur	N/A	P	N/I
6	24	M	Rt Chest wall	N/A	N	N/I
7	14	M	Lt Femur	CDB	N	N/I
8	24	M	Rt Femur	N/A	P	N/I
9	66	M	Rt Femur	N/A	P	N/I
10	19	M	Rt Femur	Osteoclastic type	P	Left scapula
11	14	F	Lt Clavicle	CDB	N	N/I
12	25	M	Rt Femur	Spindle cells with necrosis Osteoid+	N	N/I
13	28	F	Lt Humerus	MMT	N	N/I
14	11	M	Lt Femur	MMT	N	N/I
15	35	F	Lt Tibia	MMT	P	N/I
16	8	F	Lt Femur	N/A	P	N/I
17	8	F	Rt Femur	N/A	P	N/I
18	24	M	Lt Femur	N/A	N	N/I
19	16	M	Lt Tibia	N/A	N	N/I
20	10	F	Lt Fibula	N/A	N	N/I
21	17	M	Lt Fibula	MMT	N	N/I
22	24	M	Rt Jaw	Spindle cells (freq. mitosis and high labelling index, MIB-I)	N	N/I

F: female, M: male, Rt: right, Lt: left, N/A: not available, CDB: chondroblastic differentiation, MMT: malignant mesenchymal tumor, P: positive, N: negative, N/I: not involved.

**Table 3:** Showing the individual uptake values in case of both the ^18^F-FDG-PET/CT and ^99m^Tc-DMSA (V).

	^18^F-FDG-PET-CT		^ 99m^Tc-DMSA (V)			
Patient no.	Primary site SUV_max_	Lung mets SUV_max_	T/NT_max_ primary site	T/NT_avg_ primary site	Lung mets L/N_max_	Lung mets L/N_avg_
1	17.3	0.6	1.15	0.881	N	N
2	18.9	0.5	7.04	6.054	5.313	3.081
3	11.4	N	4.06	0.349	N	N
4	10.8	9.9	10.68	5.43	20.23	12.166
5	3.4	0.8	7.404	12.01	6. 214	3.633
6	6.6	N	3.64	3.72	N	N
7	5.2	N	4.89	3.743	N	N
8	4.3	1.3	5.7	4.324	2.89	2.689
9	4.1	0.5	3.098	3.226	N	N
10	5.8	0.8	6.607	3.526	N	N
11	5.7	N	4.01	3.346	N	N
12	8	N	5.65	4.986	N	N
13	2.2	N	2.19	2.06	N	N
14	5.3	N	2.596	3.508	N	N
15	15.5	2.0	3.889	3.088	1. 5	1.298
16	2.3	0.9	2.032	3.138	2.0	1.967
17	2.6	1.6	1.52	2.885	1.788	1.538
18	4.7	N	3.52	2.973	N	N
19	7.8	N	2.667	4.958	N	N
20	4	N	5.788	4.599	N	N
21	4.3	0.6	3.536	2.947	N	N
22	6.1	N	6.121	2.985	N	N
